# The Mediating Effect of Depression on the Relationship between Loneliness and Substance Use in Korean Adolescents

**DOI:** 10.3390/bs14030241

**Published:** 2024-03-17

**Authors:** Hyesun Kim

**Affiliations:** Department of Nursing, Joongbu University, Chungnam 32713, Republic of Korea; twins815@hanmail.net

**Keywords:** substance use, loneliness, depression, adolescents

## Abstract

Substance use among adolescents is a major emerging health problem worldwide. Although loneliness and depression are major risk factors for substance use, few studies have examined the relationship between loneliness, depression, and substance use in adolescents. This study aimed to determine the mediating effect of depression on the relationship between loneliness and substance use among Korean adolescents, based on the data from 53,310 adolescents from the 17th Korea Youth Risk Behavior Web-based Survey in 2021. Using a complex sample analysis module, hierarchical logistic regression analysis was employed to confirm the mediating effect of depression on the relationship between loneliness and substance use. The results showed that loneliness and depression have a significant effect on substance use (smoking, drinking alcohol, and drug use). Depression was found to have a partial mediating effect on the relationship between loneliness and substance use. Overall, the results suggested that loneliness and depression in adolescents increase substance use, and loneliness can affect substance use through depression. Therefore, proactive strategies to prevent and reduce loneliness and depression in adolescents can be effective in preventing substance use.

## 1. Introduction

Substance use is defined as the use of harmful recreational substances, such as tobacco, alcohol, and illicit drugs. Materializing into a global concern, it is particularly high in adolescence and associated with higher morbidity and mortality among teens [1,2,3,4]. Substance use that begins at a young age increases dependence and further strengthens substance use [5]. This, in turn, becomes a strong predictor of future substance use [2] and is linked to violent and aggressive behavior, social adjustment problems, crime, and mental health problems [2]. Additionally, adolescence is an important neural developmental period characterized by brain development, but repeated substance use can damage brain development [3,6]. Therefore, the risk factors of substance abuse must be identified, and a systematic and multifaceted strategy must be implemented to prevent substance abuse among Korean adolescents.

Substance use is associated with various negative emotions, one of which is loneliness [4,7]. Adolescence is a time when the importance of interpersonal relationships increases [8]; in contrast, loneliness is common in adolescence due to deficient social skills and substantial changes in social roles and relationships [9]. Loneliness has several negative effects on adolescents [8,9,10,11]. It is associated with academic obstacles, such as maladjustment to school life and poor academic achievements [9,12]; emotional problems, such as depression, increased suicide risk, and the persistence of loneliness into adulthood [9,11,12]; increased physical issues, such as increased morbidity, and early mortality [9]; and increased substance use, such as smoking, drinking, and drug use [8,9]. Psychological distress resulting from loneliness can make adolescents more vulnerable to substance use [9]. In particular, social distancing (due to the COVID-19 pandemic) further reduced social contact among adolescents and exacerbated their loneliness, leading to greater concerns about substance consumption [10].

Depression is another psychological factor that affects adolescents’ substance use [1,6]. In particular, adolescents are more likely to use substances to control negative emotions because of their high tendency toward emotional arousal and sensation-seeking [6]. McKenzie et al. [13] conducted a longitudinal study and found that nicotine dependence among adolescents with depression was two times higher than that among the general population, suggesting that depression is an important predictor of substance use. Additionally, depression has long been associated with loneliness. Frequent or long-term loneliness adversely affects mental health, resulting in anxiety and depression [11]. Stickley et al. [9] proposed that depression mediates the relationship between loneliness and substance use. However, evidence for this remains unclear, with no related studies on adolescents.

Therefore, this study aimed to identify the levels of loneliness, depression, and substance use in Korean adolescents, assess the mediating effect of depression on the relationship between loneliness and substance use, and provide basic data for future research directions and educational intervention programs.

## 2. Materials and Methods

### 2.1. Design and Study Population

This study used nationwide data from the 17th Korea Youth Risk Behavior Web-based Survey (KYRBWS) [14] to explore the mediating effect of depression on the relationship between loneliness and substance use in Korean adolescents. The KYRBWS investigates health behaviors among middle and high school students aged 12 to 18 years. Raw data for the 17th wave were collected from August to October 2022. In total, 54,848 students participated in the 17th KYRBWS; this study used data from 53,310 participants, after excluding 1538 participants because of missing responses to the question “Please indicate if anyone in your family currently smokes”.

### 2.2. Measurements

#### 2.2.1. Loneliness

Loneliness was assessed using the question, “During the past 12 months, how often have you felt lonely?” The original response scale (never, rarely, sometimes, often, and always) was reclassified into “No” (“never” to “sometimes”) and “Yes” (“often” and “always”) [8,15].

#### 2.2.2. Substance Use

Smoking was assessed using the following question for each type of cigarette: “During the past 30 days, on how many days did you smoke at least one cigarette (regular cigarette, liquid electronic cigarette, or cigarette-type electronic cigarette)?” A response of “more than one day” for any type of cigarette indicated smoking status. Drinking alcohol was assessed using the following questions: “Have you ever had more than one drink?” “During the past 30 days, on how many days did you drink more than one glass of alcohol?” and “What was the average amount of alcohol you consumed in the last 30 days?” Alcohol consumption was classified as non-drinking, non-binge-drinking, and binge-drinking. Binge-drinking was defined as drinking more than three to four drinks at a time for women and more than five drinks for men, regardless of the type of alcohol [16]. Drinking less than the abovementioned amounts or not drinking alcohol within the past 30 days was classified as non-binge-drinking [16]. Drug use was assessed using the following question: “Except for therapeutic purposes, have you ever habitually taken drugs (antianxiety drugs, stimulants, sedative-hypnotics, appetite suppressants, or opioids) or inhaled glue (cannabis, cocaine, or butane gas)?” The responses were categorized as “yes” or “no”, as per the original response scale.

#### 2.2.3. Depression

Depression was evaluated using the question, “During the past 12 months, have you felt sad or hopeless enough to stop your daily life for two weeks?” Responses were classified as either “yes” or “no”, as per the original response scale.

#### 2.2.4. Participants’ General Characteristics and Smoking- and Drinking-Related Variables

The general characteristics of the participants included gender, school, residential area, economic status, academic achievement, living with family, perceived health status, sleep satisfaction, stress, and sexual intercourse. Smoking-related variables included exposure to smoking advertisements and anti-smoking campaigns, exposure to secondhand smoke, experience of smoking cessation education, and smoking in family and close friends. Drinking-related variables included experience of alcohol education, allowing drinking at home, and exposure to alcohol advertisements in the past 30 days. The general characteristics considered for analysis are presented in Table 1 [17].

### 2.3. Data Analysis

Sampling for the KYRBWS involved population stratification, sampling distribution, and stratified cluster sampling. The data collected were investigated using the guidelines provided by the KYRBWS for complex sample analysis, and strata, cluster, and weights were considered [17]. Participants’ general characteristics, loneliness, depression, and substance use were evaluated using descriptive statistics. The Rao–Scott χ^2^ test was used to analyze differences in substance use based on participants’ general characteristics, loneliness, and depression. Baron and Kenny’s [18] hierarchical logistic regression analysis was used to assess the mediating effect of depression on the relationship between loneliness and substance use. In the Rao–Scott χ^2^ test, statistically significant variables were included as covariates in the logistic analysis (*p* < 0.05). Baron and Kenny [18] outlined the following procedure to assess the mediating effect: Step 1: Assess the effect of the independent variable on the parameter. Step 2: Assess the effect of the parameter on the dependent variable. Step 3: Simultaneously assess the influence of the independent variable and parameter on the dependent variable. All statistical analyses were conducted using IBM SPSS 25.0 (IBM, Armonk, New York, NY, USA).

### 2.4. Ethical Considerations

This study used raw data from the KYRBWS with approval from the Korea Center for Disease Control and Prevention. It was approved for exemption from review by the Institutional Review Board (IRB) of Joongbu University (Approval No. JIRB-202308100-01-230818).

## 3. Results

### 3.1. General Characteristics, Loneliness, Depression, and Substance Use

Participants’ general characteristics, loneliness, depression, and substance use are shown in Table 2. Among the participants, 15.9% felt lonely, 5.1% smoked, 10.7% drank alcohol, 0.7% used drugs, and 26.7% were depressed.

### 3.2. Differences in Substance Use according to General Characteristics, Loneliness, and Depression

Table 3 shows the differences in substance use according to participants’ general characteristics, loneliness, and depression. Smoking differed significantly depending on gender, school, residential area, economic status, academic achievement, living with family, perceived health status, sleep satisfaction, stress, sexual intercourse, exposure to smoking advertisements, exposure to secondhand smoke, experience of smoking cessation education, and smoking in family and close friends. Drinking alcohol differed significantly according to gender, school, residential area, economic status, academic achievement, living with family, perceived health status, sleep satisfaction, stress, sexual intercourse, experience of alcohol education, and allowing drinking at home. Drug use differed significantly according to economic status, academic achievement, living with family, perceived health status, sleep satisfaction, stress, and sexual intercourse. In addition, there were significant differences in smoking, drinking alcohol, and drug use based on loneliness and depression.

### 3.3. Mediating Effect of Depression on the Relationship between Loneliness and Substance Use

To determine the mediating effect of depression on the relationship between loneliness and substance use, a three-step logistic regression analysis was conducted based on the procedure suggested by Baron and Kenny [18]. Before testing the mediation effect, the assumptions of the regression analysis were tested by dummying the variables, and the Durbin–Watson statistic was found to be 1.892–1.995, indicating no autocorrelation between residuals. As a result of confirming multicollinearity, the tolerance limits were 0.276–0.969, which were all less than 1.0, and the variance expansion coefficients for all variables were 1.018–3.621, which were less than 10, indicating that there was no issue with the multicollinearity.

**Figure 1:**
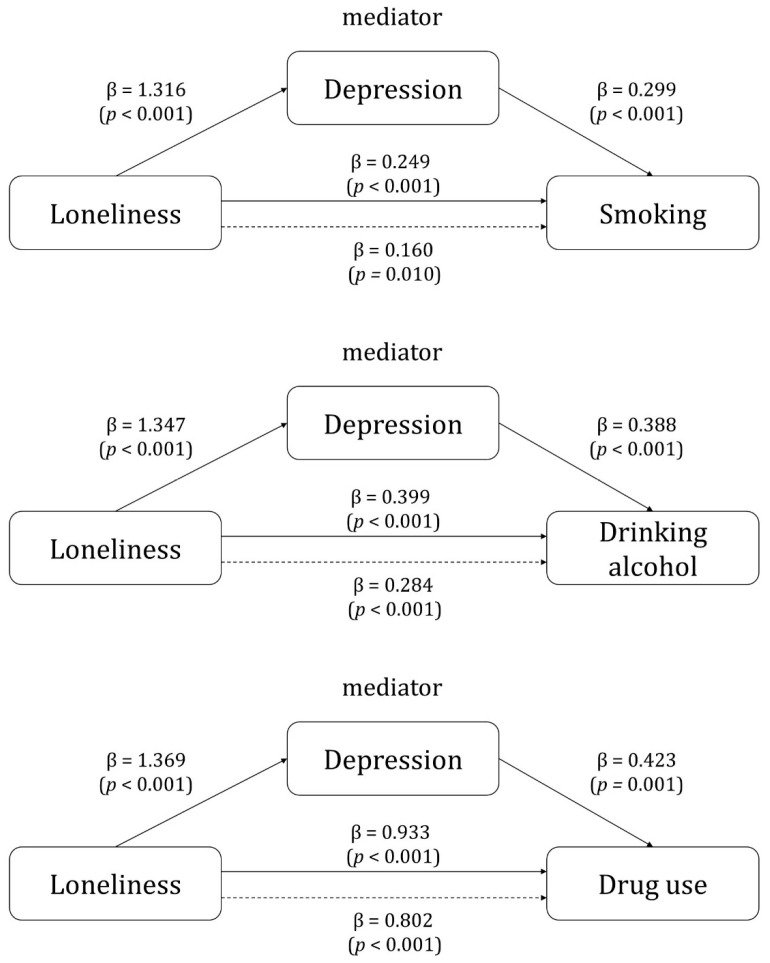
Mediating effects of depression between loneliness and substance use. Figure 1 shows the mediating effect of depression between loneliness and the use of each type of substance. The solid line is the direct effect and the dotted line is the indirect effect.

In Step 1 of the analysis, the significant effect of loneliness (independent variable) on depression (parameter) was identified (smoking: β = 1.316, *p* < 0.001; drinking alcohol: β = 1.347, *p* < 0.001; drug use: β = 1.369, *p* < 0.001). In Step 2, the results of a logistic regression analysis with loneliness as the independent variable and substance use as the dependent variable confirmed the significant effect of loneliness on substance use (smoking: β = 0.249, *p* < 0.001; drinking alcohol [binge-drinking]: β = 0.399, *p* < 0.001; drug use: β = 0.933, *p* < 0.001). In Step 3, the independent variable of loneliness and the mediating variable of depression were input as independent variables to confirm their effect on the dependent variable of substance use. Loneliness (smoking: β = 0.160, *p* = 0.010; drinking alcohol [binge-drinking]: β = 0.284, *p* < 0.001; drug use: β = 0.802, *p* < 0.001) and depression (smoking: β = 0.299, *p* < 0.001; drinking alcohol [binge alcohol]: β = 0.388, *p* < 0.001; drug use: β = 0.423, *p* = 0.001) had a significant effect on substance use. In addition, the β value of loneliness in Step 3 was lower than that in Step 2, indicating that depression had a partial mediating effect on the relationship between loneliness and substance use (Table 4, Figure 1).

## 4. Discussion

This study identified the mediating effect of depression on the relationship between loneliness and substance use among Korean adolescents, using the 17th KYRBWS data. The results showed that 15.9% of the participants aged 12 to 18 answered “Yes” to questions about loneliness, which was higher than that reported previously. Stickley et al.’s [9] study on Russian and American adolescents aged 12 to 17 found that 6.7–8.9% of boys and 14.4–14.7% of girls experienced loneliness. In this study, the relatively high level of loneliness could be explained by the fact that Koreans are more aware of loneliness due to their collectivist characteristics [19], and that during the COVID-19 pandemic, most schools converted from in-person classes to online classes, leading to social isolation and reduced outdoor activities and opportunities for social interaction [10].

Depression was reported by 26.7% of adolescents, higher than the 22.2% reported by Cena et al. [10] among Italian high school students. Although depression in Korean adolescents has decreased (28.2% in 2019), it remains the most common mental health problem, and high suicide rates due to depression remain a serious public health issue since 2011 [20]. In fact, more than half the adolescents were reported to have had depression at the time of suicide [21].

In this study, 5.1%, 10.7%, and 0.7% of the participants reported smoking, drinking, and drug use, respectively; furthermore, depression showed a partial mediating effect on the relationship between loneliness and the use of each substance. These results indicate that loneliness has a direct effect on the use of each substance and an indirect effect through depression. In other words, even if the level of loneliness is the same, the effect of loneliness on substance use may vary depending on the level of depression. This indicates that preventive strategies should target both loneliness and depression to prevent or reduce substance use among adolescents.

There is substantial evidence for the impact of loneliness on substance use in adolescents. Adolescents who felt lonely were at 1.75, 1.80, and 1.55 times higher risk of smoking [22], alcohol consumption [9], and habitual drug use [8], respectively, than those who felt less lonely. A study by Ingram et al. [4] showed that participants relied on substance use because they had no other way of escaping periods of loneliness or loneliness-related pain. The self-medication hypothesis states that individuals with mental health problems begin using substances to self-treat the symptoms associated with their illness, and explains the mechanism between loneliness and substance use [23,24]. In other words, lonely people are attracted to the psychopharmacological properties of cigarettes, alcohol, and other drugs that increase positive emotions [24,25]. They may also consume substances for the purpose of receiving recognition from peers and socializing [4,25]. This can lead to addiction, which, in turn, can lead to interpersonal conflict with others, reduced bonds, and behavioral patterns of consuming substances alone, further aggravating loneliness over time [4]. Loneliness also increases self-destructive tendencies, such as unhealthy behaviors (e.g., smoking, drinking, and drug use) [7], because it makes controlling impulses more difficult [26].

Furthermore, loneliness influences substance use via depression. The lack or loss of social relationships leads to social pain, such as loneliness, and social pain leads to depression, decreased self-control, and low self-esteem [12,27,28]. Therefore, loneliness can lead to depression, which, in turn, can lead to substance consumption. This is consistent with a study by Horigian et al. [29] that reported a mediating effect of depression on the relationship between loneliness and drug use in adults. There is considerable evidence supporting these mechanisms [29]. A meta-analysis by Groenman et al. [23] reported that internalization disorders, such as depression and anxiety in adolescents, are associated with increased substance use, especially in individuals with depression, with a 2.20-fold increase in the risk of developing substance-related disorders [23].

Depression leads to a defective stimulus barrier, resulting in a reduced ability to process emotions, eventually making individuals vulnerable to substance use [24]. When depressed individuals begin to consume substances, they try to resolve their psychological changes through repeated substance use, which makes them more physically and mentally vulnerable [8]. This may be due to the mood-altering effects of the neurotransmitter serotonin, which acts as an antidepressant [1,5,25], released as a result of exposure to chemicals such as nicotine during substance consumption. [1,5,25]. People with depression experience mood-enhancing effects to a higher degree than healthy people, and therefore, they may consume substances to overcome pleasure deficits [1,5,25]. Thus, individuals with depression may consume substances as a coping mechanism, similar to lonely individuals; however, this effect does not last long and tends to worsen depressive symptoms [30]. This further increases the risk of becoming dependent on substances [30], which can lead to habitual substance consumption, thereby creating a vicious cycle [8].

Mental health problems and substance use also affect each other [31], increasing the risk of co-occurrence, and the prognosis is worse for comorbidities [32]. Although there is still no consensus as to whether depression increases substance consumption or whether substance consumption increases depression, evidence that depression precedes substance consumption is stronger, and longitudinal studies have shown this [28,33]. In addition, while loneliness and depression have different definitions, low social skills and a negative impact on interpersonal relationships are common to both; they are not only closely related but often occur simultaneously [34]. Hence, it is necessary to develop effective intervention programs to identify each potential sign by considering the association with the relationship between them.

This study revealed that depression has a mediating effect on the relationship between loneliness and substance use. Preventive intervention is paramount because substance use not only increases individual, economic, and social costs [23] but also tends to accompany other substance use, which can lead to more serious problems [32].

The study has the following limitations. First, as secondary data collected through self-reporting were used, recall bias may have occurred. Second, due to the limitations of the database, depression and loneliness were measured as experiences over the past 12 months, but substance use was measured in the past 30 days, or no time period was given. Additionally, the results of this study should be interpreted with caution as the intended variables may not have been measured accurately; this study used a single question with dichotomous response categories and did not use validated instruments. Third, due to the cross-sectional nature of the study, caution is needed in interpreting causal relationships between the variables. Fourth, the survey data pertained to the COVID-19 pandemic period and may have reflected the special circumstances of the pandemic, such as increased loneliness or depression due to social distancing.

Nevertheless, this study is not only highly representative of the Korean adolescent population, utilizing nationwide data from the KYRBWS, but it also contributes to the literature on the mediating effect of depression on the relationship between loneliness and substance consumption in adolescents.

## 5. Conclusions

This study found that loneliness and depression are factors that influence substance use in adolescents and confirmed the partial mediating effect of depression on the relationship between loneliness and substance use. Therefore, strategies to reduce loneliness and depression and improve the formation of social relationships and coping skills should be developed to prevent substance use in Korean adolescents. Additionally, educational intervention programs to prevent loneliness and depression as part of national health policies may help prevent substance consumption among adolescents.

Future studies should examine the relationship between various mental health problems and substance consumption in adolescents, as well as between loneliness and depression. As this study was cross-sectional in nature, a longitudinal study should be conducted to clarify the direction of the relationships between the variables.

## Figures and Tables

**Table 1:** Assessment of general characteristics, smoking-related variables, and drinking-related variables.

Variables	Assessment
General characteristics	
Age	In which year and month were you born?
Gender	Male or female
School	1st, 2nd, or 3rd year of middle school or high school
Residential area ^†^	Metropolis, mid-sized city, or rural area
Economic status	What is your household’s financial situation? Possible responses are high, middle-high, middle, middle-low, or low.
Academic achievement	How has your academic performance been in the past 12 months? Possible responses are high, middle-high, middle, middle-low, or low.
Living with family	What is your current living situation? Possible responses are living with family, living with relatives, boarding house/living alone, dormitory, or youth facilities.
Perceived health status	How do you perceive your usual health status? Possible responses are very healthy, healthy, fair, unhealthy, or very unhealthy.
Sleep satisfaction	In the last 7 days, did you have enough sleep time to recover from fatigue? Possible responses are very sufficient, sufficient, fair, insufficient, or very insufficient.
Stress	How much stress do you usually feel? Possible responses are very high, high, moderate, low, or none.
Sexual intercourse	Have you ever had sexual intercourse? Yes or no
Smoking-related variables	
Exposure to smoking advertisements	In the last 30 days, have you seen any smoking advertisements? Possible responses are magazines, internet, convenience store, supermarket, or not seen.
Exposure to anti-smoking campaigns	In the last 12 months, have you seen or heard any anti-smoking campaigns? Possible responses are TV, radio, TV program/news, internet, newspaper, subway/bus stop, or not seen.
Exposure to secondhand smoke	During the last 7 days, on how many days did you inhale secondhand smoke at home, school, or indoors? Possible responses are between 0 and 7 days per week.
Experience of smoking cessation education	In the last 12 months, have you received smoking prevention or cessation education at school? Yes or no
Smoking in family	Please indicate if anyone in your family currently smokes. Possible responses are none, father, mother, siblings, or grandparents
Smoking in close friends	Do you have any close friends who smoke? Possible responses are none, a few, many, or everyone
Drinking-related variables	
Experience of alcohol education	In the last 12 months, have you received any alcohol education at school? Yes or no
Allowing drinking at home	Have your parents or relatives ever encouraged (allowed) you to drink alcohol at home? Yes or no
Exposure to alcohol advertisements	In the last 30 days, on how many days have you seen alcohol advertisements on the television, internet, social media, posters, and so on? Possible responses are 1 day/month, 2~3 days/month, 1~2 days/week, 3~4 days/week, 5~6 days/week, 7 days, or not seen.

Note: ^†^ Categorized by sampling design.

**Table 2:** General characteristics, loneliness, depression, and substanc use (N = 53,310).

Variable	Categories	n ^†^ (%) ^‡^ or M ± SD ^‡^
Independent variable		
Loneliness	No	44,837 (84.1)
	Yes	8473 (15.9)
Dependent variables		
Smoking	Yes	2666 (5.1)
	No	50,644 (94.9)
Drinking alcohol	Binge-drinking	2534 (4.9)
	Non-binge-drinking	14,896 (28.0)
	Non-drinking	35,880 (67.1)
Drug use	Yes	384 (0.7)
	No	52,926 (99.3)
Mediating variable		
Depression	Yes	14,239 (26.7)
	No	39,071 (73.3)
General characteristics		
Age		15.24 ± 0.02
Gender	Male	27,644 (51.7)
	Female	25,666 (48.3)
School	Middle school	29,064 (50.7)
	High school	24,246 (49.3)
Residential area	Metropolis	26,701 (49.9)
	Mid-sized city	23,487 (45.6)
	Rural area	3122 (4.5)
Economic status	High	21,067 (40.3)
	Middle	26,226 (48.8)
	Low	6017 (10.9)
Academic achievement	High	20,028 (37.3)
	Middle	16,433 (31.0)
	Low	16,849 (31.7)
Living with family	Yes	50,990 (96.2)
	No	2320 (3.8)
Perceived health status	Unhealthy	4860 (9.2)
	Fair	13,848 (26.0)
	Healthy	34,602 (64.8)
Sleep satisfaction	Sufficient	12,480 (22.9)
	Fair	17,393 (32.4)
	Insufficient	23,437 (44.7)
Stress	High	20,598 (38.7)
	Moderate	22,592 (42.6)
	Low	10,120 (18.7)
Sexual intercourse	Yes	2759 (5.4)
	No	50,551 (94.6)
Smoking-related variables		
Exposure to smoking advertisements	Yes	18,147 (33.8)
	No	35,163 (66.2)
Exposure to anti-smoking campaigns	Yes	38,678 (72.7)
	No	14,632 (27.3)
Exposure to secondhand smoke	Yes	28,354 (53.3)
	No	24,956 (46.7)
Experience of smoking cessation education	Yes	34,508 (63.1)
	No	18,802 (36.9)
Smoking in family	Yes	28,069 (51.6)
	No	25,241 (48.4)
Smoking in close friends	Yes	16,375 (31.2)
	No	36,935 (68.8)
Drinking-related variables		
Experience of alcohol education	Yes	18,103 (32.9)
	No	35,207 (67.1)
Allowing drinking at home	Yes	17,903 (33.8)
	No	35,407 (66.2)
Exposure to alcohol advertisements	<1/week	27,611 (51.5)
	≥1/week	25,699 (48.5)

Note: ^†^ Unweighted; **^‡^** weighted. SD = Standard deviation.

**Table 3:** Differences in substance use according to general characteristics, loneliness, and depression (N = 53,310).

Variable	Categories	Smoking		Rao–Scott χ^2^ Test or t	*p*	Drinking Alcohol			Rao–Scott χ^2^ Test or t	*p*	Drug Use		Rao–Scott χ^2^ Test or t	*p*
		Yes	No			Binge-Drinking	Non-Binge-Drinking	Non-Drinking			Yes	No		
		n ^†^ (%) ^‡^ or M ± SD ^‡^	n ^†^ (%) ^‡^ or M ± SD ^‡^			n ^†^ (%) ^‡^ or M ± SD ^‡^	n ^†^ (%) ^‡^ or M ± SD ^‡^	n ^†^ (%) ^‡^ or M ± SD ^‡^			n ^†^ (%) ^‡^ or M ± SD ^‡^	n ^†^ (%) ^‡^ or M ± SD ^‡^		
Loneliness	No	1950 (72.9)	42,887 (84.7)	271.13	<0.001	1789 (70.6)	11,930 (80.0)	31,118 (86.7)	345.21	<0.001	223 (55.9)	44,614 (84.3)	228.99	<0.001
	Yes	716 (27.1)	7757 (15.3)			745 (29.4)	2966 (20.0)	4762 (13.3)			161 (44.1)	8312 (15.7)		
Depression	Yes	1184 (44.2)	13,055 (25.8)	385.42	<0.001	1182 (47.1)	4810 (32.0)	8247 (23.0)	412.20	<0.001	196 (52.3)	14,043 (26.5)	130.32	<0.001
	No	1482 (55.8)	37,589 (74.2)			1352 (52.9)	10,086 (68.0)	27,633 (77.0)			188 (47.7)	38,883 (73.5)		
Age		16.30 ± 0.04	15.18 ± 0.02	24.16	<0.001	16.46 ± 0.03	15.77 ± 0.02	14.93 ± 0.02	1045.49	<0.001	15.48 ± 0.09	15.64 ± 0.02	1.78	0.075
Gender	Male	1826 (69.7)	25,818 (50.7)	197.93	<0.001	1373 (56.0)	8885 (59.7)	17,386 (48.1)	144.86	<0.001	227 (56.2)	27,417 (51.7)	3.03	0.082
	Female	840 (30.3)	24,826 (49.3)			1161 (44.0)	6011 (40.3)	18,494 (51.9)			157 (43.38)	25,509 (48.3)		
School	Middle school	668 (22.3)	28,396 (52.3)	499.57	<0.001	529 (17.8)	6187 (37.0)	22,348 (58.9)	872.95	<0.001	188 (46.8)	28,876 (50.8)	2.38	0.124
	High school	1998 (77.7)	22,248 (47.7)			2005 (82.2)	8709 (63.0)	13,532 (41.1)			196 (53.2)	24,050 (49.2)		
Residential area	Metropolis	1214 (46.3)	25,487 (50.1)	3.88	0.026	1070 (43.3)	6956 (46.8)	18,675 (51.8)	20.66	<0.001	185 (46.1)	26,516 (50.0)	2.22	0.111
	Mid-sized city	1289 (49.2)	22,198 (45.4)			1272 (51.3)	6882 (47.9)	15,333 (44.2)			178 (50.4)	23,309 (45.6)		
	Rural area	163 (4.5)	2959 (4.5)			192 (5.4)	1058 (5.3)	1872 (4.0)			21 (3.5)	3101 (4.4)		
Economic status	High	955 (36.8)	20,112 (40.5)	93.01	< 0.001	891 (36.3)	5397 (36.9)	14,779 (42.1)	93.08	<0.001	151 (39.0)	20,916 (40.3)	16.52	<0.001
	Middle	1198 (44.1)	25,028 (49.1)			1170 (45.8)	7396 (49.5)	17,660 (48.7)			156 (41.1)	26,070 (48.8)		
	Low	513 (19.1)	5504 (10.4)			473 (17.9)	2103 (13.6)	3441 (9.2)			77 (19.9)	5940 (10.8)		
Academic achievement	High	586 (21.8)	19,442 (38.1)	260.62	<0.001	667 (26.1)	4696 (31.3)	14,665 (40.6)	186.60	<0.001	154 (38.8)	19,874 (37.3)	9.27	<0.001
	Middle	674 (25.4)	15,759 (31.3)			675 (27.4)	4583 (30.9)	11,175 (31.3)			88 (21.6)	16,345 (31.1)		
	Low	1406 (52.8)	15,443 (30.6)			1192 (46.5)	5617 (37.8)	10,040 (28.1)			142 (39.6)	16,707 (31.7)		
Living with family	Yes	2441 (92.0)	48,549 (96.5)	65.84	<0.001	2306 (91.5)	14,145 (95.5)	34,539 (96.9)	63.88	<0.001	332 (86.8)	50,658 (96.3)	69.67	<0.001
	No	225(8.0)	2095 (3.5)			228 (8.5)	751 (4.5)	1341 (3.1)			52 (13.2)	2268 (3.7)		
Perceived health status	Unhealthy	330 (12.3)	4530 (9.0)	15.30	<0.001	310 (11.9)	1620 (11.0)	2930 (8.3)	27.58	<0.001	85 (23.5)	4775 (9.1)	47.95	<0.001
	Fair	635 (24.7)	13,213 (26.1)			624 (24.8)	3880 (26.0)	9344 (26.0)			108 (27.9)	13,740 (26.0)		
	Healthy	1701 (63.0)	32,901 (64.9)			1600 (63.3)	9396 (63.0)	23,606 (65.7)			191 (48.6)	34,411 (64.9)		
Sleep satisfaction	Sufficient	415 (16.1)	12,065 (23.3)	78.49	<0.001	371 (15.3)	2936 (19.5)	9173 (24.9)	119.97	<0.001	62 (15.5)	12,418 (23.0)	26.83	<0.001
	Fair	727 (27.0)	16,666 (32.6)			649 (24.9)	4695 (31.1)	12,049 (33.4)			90 (21.0)	17,303 (32.4)		
	Insufficient	1524 (56.9)	21,913 (44.1)			1514 (59.8)	7265 (49.4)	14,658 (41.7)			232 (63.5)	23,205 (44.6)		
Stress	High	1310 (48.8)	19,288 (38.1)	52.77	<0.001	1240 (49.1)	6415 (42.8)	12,943 (36.2)	74.89	<0.001	239 (61.2)	20,359 (38.5)	39.55	<0.001
	Moderate	945 (35.4)	21,647 (43.0)			906 (35.4)	5988 (40.3)	15,698 (44.0)			105 (27.6)	22,487 (42.7)		
	Low	411 (15.8)	9709 (18.9)			388 (15.6)	2493 (16.9)	7239 (19.8)			40 (11.2)	10,080 (18.8)		
Sexual intercourse	Yes	974 (37.1)	1785 (3.7)	5087.37	<0.001	805 (32.3)	1273 (8.8)	681 (2.0)	2270.76	<0.001	91 (25.1)	2668 (5.2)	246.98	<0.001
	No	1692 (62.9)	48,859 (96.3)			1729 (67.7)	13,623 (91.2)	35,199 (98.0)			293 (74.9)	50,258 (94.8)		
Smoking-related variables														
Exposure to smoking advertisements	Yes	1911 (71.7)	33,252 (65.9)	37.82	<0.001	-	-	-	-	-	-	-	-	-
	No	755 (28.3)	17,392 (34.1)			-	-	-						
Exposure to anti-smoking campaigns	Yes	1894 (71.1)	26,784 (72.8)	3.58	0.059	-	-	-	-	-	-	-	-	-
	No	772 (28.9)	13,860 (27.2)			-	-	-						
Exposure to secondhand smoke	Yes	1849 (69.4)	26,505 (52.4)	295.38	<0.001	-	-	-	-	-	-	-	-	-
	No	817 (30.6)	24,139 (47.6)			-	-	-						
Experience of smoking cessation education	Yes	1584 (57.8)	32,924 (63.4)	32.62	<0.001	-	-	-	-	-	-	-	-	-
	No	1082 (42.2)	17,720 (36.6)			-	-	-						
Smoking in family	Yes	1729 (64.3)	26,340 (50.9)	164.72	<0.001	-	-	-	-	-	-	-	-	-
	No	937 (35.7)	24,304 (49.1)			-	-	-						
Smoking in close friends	Yes	2485 (93.3)	13,890 (27.8)	4575.01	<0.001	-	-	-	-	-	-	-	-	-
	No	181 (6.7)	36,754 (72.2)			-	-	-						
Drinking-related variables														
Experience of alcohol education	Yes	-	-	-	-	748 (28.6)	4699 (30.4)	12,656 (34.3)	39.69	<0.001	-	-	-	-
	No					1786 (71.4)	10,197 (69.6)	23,224 (65.7)						
Allowing drinking at home	Yes	-	-	-	-	1613 (63.2)	8711 (58.7)	7579 (21.2)	3571.29	<0.001	-	-	-	-
	No					921 (36.8)	6185 (41.3)	28,301 (78.8)						
Exposure to alcohol advertisements	< 1/week	-	-	-	-	1370 (53.9)	7782 (51.8)	18,459 (51.2)	3.70	0.026	-	-	-	-
	≥ 1/week					1164 (46.1)	7114 (48.2)	17,421 (48.8)						

Note: ^†^ Unweighted; ^‡^ weighted. SD = Standard deviation.

**Table 4:** The mediating effect of depression on the relationship between loneliness and substance use (N = 53,310).

Variables	Step	Independent Variables	Dependent Variables	B	SE	AOR	*p*	Model Fit	Nagelkerke R^2^	Sobel Test
								Wald F (*p*)		Z (*p*)
Smoking ^†^	1	Loneliness	Depression	1.316	0.029	3.727	<0.001	357.32 (<0.001)	0.287	5.30 (<0.001)
	2	Loneliness	Smoking	0.249	0.060	1.283	<0.001	174.81 (<0.001)	0.378	
	3	Loneliness	Smoking	0.160	0.062	1.174	0.010	168.75 (<0.001)	0.380	
		Depression	Smoking	0.299	0.056	1.349	<0.001			
Drinking alcohol ^‡^	1	Loneliness	Depression	1.347	0.029	3.845	<0.001	399.57 (<0.001)	0.276	13.24 (<0.001)
	2	Loneliness	Drinking alcohol	0.729	0.057	2.073	<0.001	232.60 (<0.001)	0.293	
	3	Loneliness	Drinking alcohol	0.492	0.060	1.636	<0.001	228.36 (<0.001)	0.298	
		Depression	Drinking alcohol	0.801	0.058	2.228	<0.001			
Drug use ^∥^	1	Loneliness	Depression	1.369	0.029	3.933	<0.001	632.61 (<0.001)	0.272	3.64 (<0.001)
	2	Loneliness	Drug use	0.933	0.124	2.543	<0.001	39.18 (<0.001)	0.093	
	3	Loneliness	Drug use	0.802	0.128	2.230	<0.001	36.57 (<0.001)	0.096	
		Depression	Drug use	0.423	0.116	1.527	0.001			

Note 1: ^†^ Adjusted for age, gender, school, residential area, economic status, academic achievement, living with family, perceived health status, sleep satisfaction, stress, sexual intercourse, exposure to smoking advertisements, exposure to secondhand smoke, experience of smoking cessation education, smoking in family, and smoking in close friends; ^‡^ adjusted for age, gender, school, residential area, economic status, academic achievement, living with family, perceived health status, sleep satisfaction, stress, sexual intercourse, experience of alcohol education, allowing drinking at home, and exposure to alcohol advertisements; ^∥^ adjusted for economic status, academic achievement, living with family, perceived health status, sleep satisfaction, stress, and sexual intercourse. Note 2: ^‡^ Results of binge-drinking compared to non-drinking using multinomial logistic analysis; AOR = adjusted odds ratio.

## Data Availability

Data were obtained from the Korea Disease Control and Prevention Agency (KDCA) and are available from https://www.kdca.go.kr/yhs/ (accessed on 1 September 2023).
